# Leaching of Copper Contained in Waste Printed Circuit Boards, Using the Thiosulfate—Oxygen System: A Kinetic Approach

**DOI:** 10.3390/ma15072354

**Published:** 2022-03-22

**Authors:** Eleazar Salinas-Rodríguez, Juan Hernández-Ávila, Eduardo Cerecedo-Sáenz, Alberto Arenas-Flores, Maria A. Veloz-Rodríguez, Norman Toro, Maria del P. Gutiérrez-Amador, Otilio A. Acevedo-Sandoval

**Affiliations:** 1 Academic Area of Earth Science and Materials, Institute of Basis Sciences and Engineering, Autonomous University of the State of Hidalgo, Highway Pachuca-Tulancingo km. 4.5, Mineral de la Reforma 42184, Hidalgo, Mexico; herjuan@uaeh.edu.mx (J.H.-Á.); mardenjazz@yahoo.com.mx (E.C.-S.); arenasa@uaeh.edu.mx (A.A.-F.); mveloz@uaeh.edu.mx (M.A.V.-R.); 2 Faculty of Engineering and Architecture, Universidad Antonio Prat, Iquique 1100000, Chile; notoro@unap.cl; 3 Apan High School, Autonomous University of the State of Hidalgo, Highway Apan-Calpulalpan km. 8, Apan 43920, Hidalgo, Mexico; gutierrezam@yahoo.com; 4 Academic Area of Chemistry, Institute of Basis Sciences and Engineering, Autonomous University of the State of Hidalgo, Highway Pachuca-Tulancingo km. 4.5, Mineral de la Reforma 42184, Hidalgo, Mexico

**Keywords:** copper, printed circuit boards, leaching, kinetics, thiosulfate, WEEE

## Abstract

The present work is related to the treatment of crushed waste of printed circuit boards (WPCBs) from electrical and electronic devices (WEEE), carrying out the recovery of copper in solution. In the first stage, the studied material was characterized by AAS, SEM-EDS, and XRD. The results revealed significantly high amounts of copper (744.42 mg/g), compared with the rest of the metals present in the sample, mainly iron and zinc. In the second stage of the work, alkali dynamic leaching experiments were carried out in the $S_{2}O_{3}^{-2}-{\;O}_{2}$ medium, evaluating important kinetic variables in order to verify the controlling step of the system and adjust the data to a kinetic model. According to the results obtained from the various experimental tests executed, it was found that in the studied system of $S_{2}O_{3}^{-2}-{\;O}_{2}$, the leaching of copper was preferably adjusted to the model of spherical particles with a shrinking core finding a mixed chemical–diffusive control, with values of E_a_ = 25.78 kJ/mol and *n* = 0.22 (for the leaching reagent), indicating that the reaction was controlled by the oxygen transport to the solid–liquid interface and also by the chemical reaction in the surface of particles, obtaining up to 99.82% copper in solution.

## 1. Introduction

The exponential technological developments of recent decades, have had, as a counterpart, the accumulation of electrical and electronic waste (WEEE), which is the fastest growing type of waste worldwide [1]. A decrease in prices, the innovation of new technologies, and the addition of new features are leading to the replacement of electrical and electronic products at a rapid rate, with lifetime estimated at 2–3 years [2]; this short technological gap is the result of a highly consuming society, which wants to satisfy its own aspirations or join a new lifestyle [3,4,5].

Once this type of technology is obsolete or rejected, the owner never stops to think what happens to the wastes, since, in addition to the positive effects that the use of electrical and electronic devices provide, there are negative effects [6,7], and when WEEE is not managed properly, it results in pollution of the environment and damage to health, since many of the materials, such as brominated and chlorinated substances, heavy metals, acids, plastics, and plastic additives, are highly toxic when not properly managed [1,8,9,10]. This has become an emerging problem of the 21st century [11,12,13,14].

A current estimate shows that almost 45 million tons of WEEE are generated globally per year and the number is growing at an exponential rate [15,16], compared to other municipal solid waste [17].

The relative composition of the WEEE is 38% ferrous metals, 28% non-ferrous metals, 19% plastics, 4% glass, 1% wood, and 10% others [12,18,19,20]. Therefore, there is an urgent need to recover the maximum amount of recyclable material from these wastes [21]. However, this issue is complex since these technological wastes are difficult to dispose of and recycling is complex and expensive [8,20,22]. Within the electrical and electronic structures, there are PCBs (printed circuit boards) that are crucial for the manufacture of about 1 trillion products. These are found in almost all electronic devices, and are even present in several weapons systems and aerospace hardware [20,21,22,23].

PCBs are considered the basis of the electrical and electronic industry, that is, they are the platform on which components such as semiconductors, chips, and capacitors are assembled providing an interconnection between them [23,24]. PCBs represent only 6% of the WEEE total weight and yet are a significant proportion of the value contained in this type of waste [19,25].

In general, PCBs contain about 30% metals. Their non-metallic fraction includes plastics, epoxy resins, and glass; the content oscillates about >70%. The typical metals consist of 20% Cu, 8% Fe, 4% Sn, 2% Ni, 2% Pb, 1% Zn, 0.2% Ag, 0.1% Au, and 0.005% Pd. Additionally the non-metallic proportion of the PCBs is based on resins and reinforced materials that can be reused as fillings in composite materials that are proposed as part of a circular economy, which has the objective of total recycling and reuse of the materials contained in these kind of wastes [6,9,12,13,17,21,26].

In addition to the special case of precious metals contained in WEEE, there is the issue of copper, which is almost ubiquitous in PCBs; economically it is the most interesting metal for recovery and use as a secondary raw material due to its relatively high content compared to the corresponding content in exploitable minerals [27]. The recovery of metals, particularly copper from PCBs as a secondary source, can support the conservation of primary sources and prevent environmental degradation while contributing to the transition towards a circular economy. In addition, the high copper concentrations reported in previous studies (in the range of 100–350 g/kg), suggest that copper recovery from these sources is of both ecological and economic interest [28].

Additionally, copper has traditionally been obtained from its minerals by pyrometallurgical methods that have the drawback of environmental pollution caused by the release of sulfur gases. Hydrometallurgical processes can be cheaper than those based on pyrometallurgical methods, because they are generally less energy-demanding and they leave a wider range of usable by-products [29].

In WEEE, copper is present in elemental form or as alloy [9]. Among its physical and chemical properties, its good ductility, high thermal conductivity, resistance to corrosion, and good electrical conductivity should be noted [17]. There are two routes to recovering copper from WEEE—hydro- or pyrometallurgy [22]. However, the hydrometallurgical process is by far the most widely applied in the recovery of metallic components from WEEE, because it is more predictable and more easily controlled [30] compared to the pyrometallurgical process.

Acidic and basic solutions are regularly used as leaching reagents in the hydrometallurgical treatment of WPCBs [31], the selection of one method or another depends on the features of the wastes. However, there are several problems that must be solved, including increasing the leaching rate and selectivity while reducing the consumption of reagent and the volume of residual solutions [23].

Many processes has been used to recover copper from WPCBs, including the use of acids, ionic liquids, combinations of pyrometallurgical processes with hydrometallurgy and electrowining, and even the use of bioleaching. Acidic leaching solutions have led to the recovery of Cu, with interesting results. Some researchers have used nitric acid and obtained recoveries of up to 99.9% without dissolution of the gold involved and also using oxidants such as ozone or H_2_O_2_ [32,33,34,35]. Others have used sulfuric and hydrochloric acids to recover Cu from WPCBs and have had adequate results [36,37,38].

Recently, some researchers have carried out work using a pyrolysis treatment followed by flotation to recover Cu from WPCBs, improving the recovery of Cu from 6.35% to 96.36% with such treatment [39]. In addition, the use of bacteria for bioleaching of Cu contained in these wastes was carried out [40]. Although this process is adequate environmentally, the reaction time is too long (about 12 days), but the recoveries are adequate (94% for Cu).

Other work has employed ammonium sulfate–H_2_O_2_ [41,42] to recover Cu from PCBs, having good results. However, all the above could have been contaminated since there has not been any study of the residues produced by these kind of processes, which would be of importance [8].

In this work, the authors propose the use of thiosulfate as leaching reagent to recover the copper contained in PCBs, as an hydrometallurgical alternative to leaching in an alkaline medium, due to its complexation capacity [43,44,45,46] and degree of stability in the compound formed, which is the copper thiosulfate [47]. Generally the formation of this compound is reported as a problem in the leaching of precious metals (Au and Ag) due to the consumption and degradation of thiosulfate that occurs, but in this case the authors have taken the advantage of this tendency to recover the copper present in these types of wastes. In addition, as the system is based in thiosulfate, oxygen has the fundamental feature of impacting the environment to a lesser degree compared to acid leaching, which is the most widely used process for the recovery of copper from oxidized minerals [17,48].

As is known, the thiosulfate–oxygen system has been shown to leach precious metals such as Au and Ag. However, no work have been carried out to leach Cu from WPCBs with the above-mentioned system, while there has been some work carried out on leaching Cu using ammonium and Cu^2+^, or ammonia–ammonium sulfate [37,42]. For this reason, the present work is of importance due principally to the use of a non-toxic reagent which will be used directly without the addition of an oxidant such as HNO_3_, H_2_O_2_, O_3_, Cu^2+^, or Fe^2+^. In this process, the quick dissolution of Cu to Cu^2+^ and its complexation with thiosulfate, can avoid the thiosulfate oxidation and therefore improving in its recovery.

## 2. Materials and Methods

The characterization made to the material of study, which was obtained from the Mexican company, was carried out by atomic absorption spectrometry (AAS) using a spectrophotometer PERKIN-ELMER model 200 Analyst, manufactured in Waltham, MA, USA (located at the UAEH), in order to determine the copper content in the sample; for this, 1 g of sample was dissolved in a concentrated HNO_3_ solution (J.T. Baker, 66.7% assay, manufactured by J.T. Baker brand Chemistry, Allentown, PA, USA), the obtained solution was decanted into a 200 mL volumetric flask and calibrated with distilled water and the dilutions and standards were prepared with identical matrices for further chemical analysis.

The result obtained from the AAS characterization showed that copper was present in majority quantities compared to the rest of the metals found, as shown in Table 1, which shows the amount of copper, iron and zinc in mg/g (mg are miligrams, and g are grams). It is worth mentioning that only one fraction of the sample evaluated corresponded to the metallic part and the other, non-metallic fraction, was composed by fiberglass [2]. It is important to point out that the company previously recovered precious metals such as Au and Ag by leaching the WPCBs using cyanide, remaining only the base metals in the residues to be further processed [49], as was done in this work.

**Figure 1:**
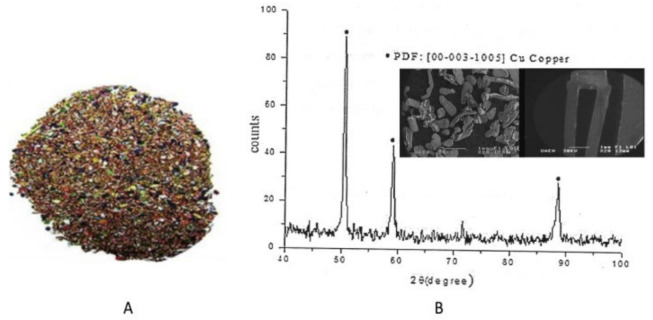
(**A**) Material used in the conducted series of experiments and (**B**) XRD spectrum and SEM (SE) images showing the morphology of the material.

In Figure 1A, an image of the material is shown. This material was used in all the experiments, as well as the XRD spectrum (Figure 1B), from which it can be seen that the peaks correspond to the PDF file (00-003-1005) for copper. Figure 1B also shows the SEM (SE) images. The irregular morphology of the particles can be observed, and bright sections are also seen. These correspond to the typical non-metallic part of the PCBs. The particle sizes ranged from 0.5 mm to 1.2 mm, which could be beneficial for the metal extraction [18].

### 2.1. Alkali Dynamic Leaching ($S_{2}O_{3}^{-2}-O_{2}$ Medium)

**Figure 2:**
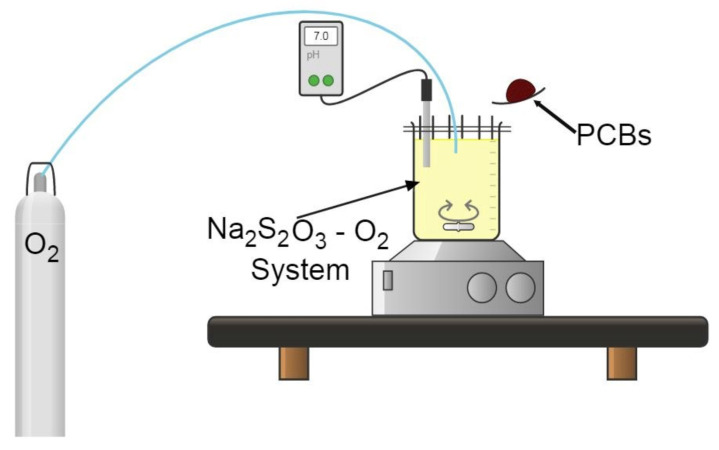
Experimental array used for the copper leaching study.

The experiments performed on the studied material were carried out in a 1 L reactor, mounted on a heating grid with automatic temperature control and magnetic stirrer. The pH of the solution was constantly measured with a pH meter (Dual Star PH/ISE with electrode and probe ATC brand Thermo Orion, manufactured by ThermoFisher Scientific, Waltham, MA, USA). For all the experiments carried out, distilled water and 5 g of sample previously sieved and bounded to the 50 mesh according the Tyler^®^ series were used. This represented a solid/liquid ratio of 1:100, as shown in Figure 2.

During the kinetic study, distilled water was used and 5 g of sample, previously sieved, was treated in a 0.5 L sodium thiosulfate pentahydrate (J.T. Baker manufactured by J.T. Baker brand Chemestry, Allentown, PA, USA) and 1 atm of oxygen partial pressure, injected into solution; the pH was kept constant by adding NaOH 2M solution (J.T. Baker, 98.6% assay manufactured by J.T. Baker brand Chemestry, Allentown, PA, USA ) to each experiment. The aliquots were taken at pre-set time ranges for further analysis by AAS. All experiments were executed over a time period of 240 min. Finally, the characterization of copper contained in the PCBs was performed using a JEOL Scanning Electron Microscope JSM-6300, manufactured by JEOL Tokyo, Japan (Located at the UAEH); likewise, it was characterized with an X-ray diffractometer Equinox, manufactured by INEL at Artenary, Centre Val de Loire, France (located at the UAEH), with a sweep time of 5 min for each sample, and to index the obtained diffractograms, the MATCH version 1.1 software (developed by Crystal Impact, Bonn, Germany) was used. Table 2 shows the experimental conditions used for this study.

### 2.2. Atomic Absorption Spectrometry

The aliquots obtained from the various leaching experiments were analyzed with a Perkin Elmer Atomic Absorption Spectrometer, model 200 Analyst, in order to assess the concentration of copper present in the leached liquor. Variations in the mass due to sampling of aliquots and the addition of pH regulator reagent, were corrected by simple mathematical calculations. The quantification of the leached copper for the kinetics study, was calculated according to the following expression:

(1) $$X_{\mathrm{Cu}}=\frac{{\mathrm{Cu}}_{\mathrm{sol}}}{{\mathrm{Cu}}_{T}}$$

where X_Cu_ is the fraction of copper in solution (from 0 to 1), Cu_sol_ is the concentration of Cu (in ppm) in the solution at determined time (t), and Cu_T_ is the total concentration of copper (in ppm).

## 3. Results

From the series of experiments carried out, the following variables were evaluated: temperature, reagent concentration, stirring rate, oxygen partial pressure, and pH, with the conditions previously described.

### 3.1. Kinetic Study of Leaching of Cu in $S_{2}O_{3}^{-2}-O_{2}$ Medium

**Figure 3:**
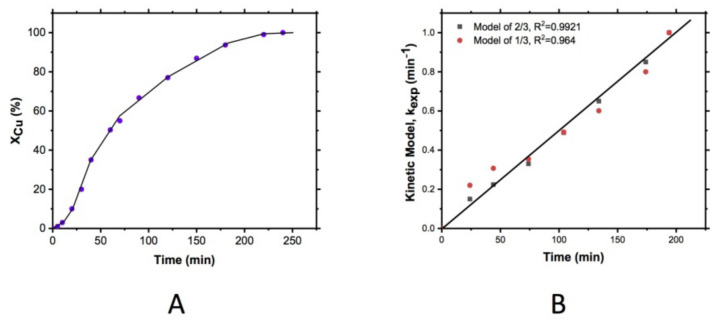
(**A**) Type S curve for the Cu leaching and (**B**) treatment of leaching data with the kinetics models of 1/3 [1 − (1 − X_Cu_)^1/3^], and 2/3 [1 − 3(1 − X_Cu_)^2/3^ + 2 (1 − X_Cu_)].

The Kinetic study of leaching of Cu contained in the WPCBs, was carried out evaluating two kinetics models to determine which of them fitted better. Figure 3A, shows the behavior of Cu extraction with respect to time. It can be seen that the extraction process comprised an induction period, followed by a progressive conversion stage and finally, the stabilization zone at the end of the reaction. On the other hand, Figure 3B shows the treatment of leaching data with both kinetic models of the shrinking core for chemical control (1/3, Equation (2)) and diffusive control (2/3, Equation (4)). It can be seen that for the progressive stage of reaction, this fitted well to the model of 2/3 for diffusive control, and according with these results, the leaching data obtained in all effects studied, were treated with this model (Equation (4)).

(2) [1−(1−XCu)13]=kexp× t

where

(3) $$k_{exp}=\frac{V_{M}k_{q}c_{A}^{n}}{r_{0}}$$

(4) [1−3(1−XCu)23+21−XCu]=kexp× t

where

(5) $$k_{exp}=\frac{2V_{M}D_{c}c_{A}}{r_{0}^{2}}$$

where X_Cu_ is the reacted fraction of Cu (from 0 to 1), V_M_ is the molar volume of material (Cu = 8.09 cm^3^/mol), c_A_ is the concentration of leaching reactant (in this case the thiosulfate in mol·L^−1^), k_q_ is the kinetic coefficient (in min^−1^), D_c_ is the diffusion coefficient through the product layer, r_0_ is the initial radius of particle (average, in m), *k_exp_* is the experimental constant (in min^−1^), t is the time (in minutes), and n is the order of the reaction.

#### 3.1.1. Effect of Reagent Concentration

**Figure 4:**
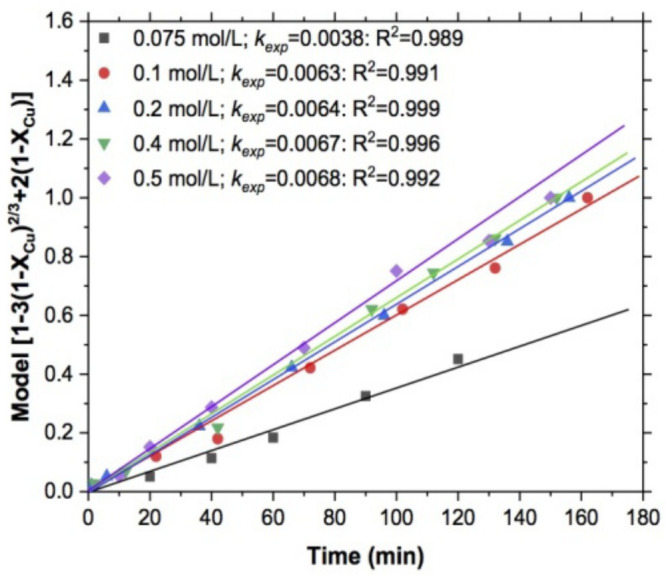
Determination of *k_exp_*, model of shrinking core, and diffusive control: effect of thiosulfate concentration.

The effect of the reagent concentration was carried out with the following experimental conditions: T = 318 K, pH = 10, $\left[S_{2}O_{3}^{-2}\right]$ = 0.075, 0.1, 0.2, 0.4, and 0.5 mol/L, stirring rate of 750 RPM, and 5 g of sample. In Figure 4, the kinetic adjustment of the variable evaluated in the range mentioned is shown. It can be noted that the *k_exp_* values increased slightly as reagent concentration also increased, as was reported by Puente-Siller et al., [50] who found that at concentrations higher than 0.1 mol/L the thiosulfate consumption was more efficient because the degradation of the products of the reaction rate with the reagent was due to the fact that the system had been saturated with oxygen in a constant manner [51] throughout all experiments evaluated.

**Figure 5:**
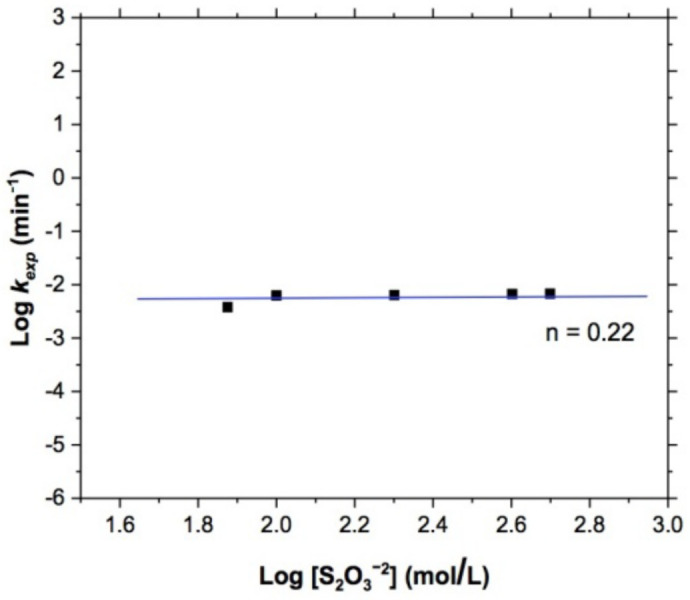
Copper leaching. Effect of the reagent concentration, order of reaction n = 0.22. Experimental conditions: 318 K, pH 10, 750 RPM, and 1 atm O_2_.

To confirm the above, it was essential to determine the reaction order of this variable, so in Figure 5, a straight line representing the *k_exp_* of the effect studied was obtained. This resulted from plotting the logarithm of *k_exp_* vs. the logarithm of the reagent concentration tested (i.e., the slope m, obtained from this plot [52,53]), the value of the reaction order calculated from the straight line showed a reaction order of the system of n = 0.22. This value indicated that the *k_exp_* was determined by a factor different from the reagent concentration, which confirmed that effectively our system was governed by the control of mass transport to the solid–liquid interface.

#### 3.1.2. Effect of the Temperature

**Figure 6:**
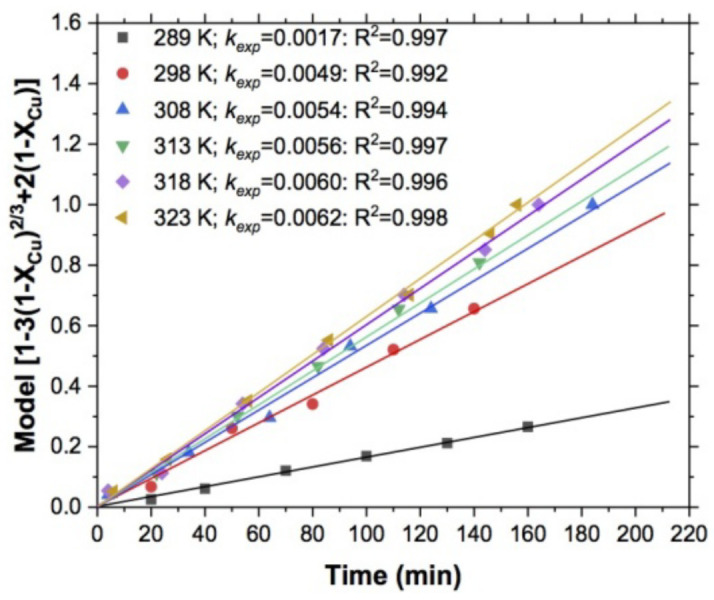
Determination of *k_exp_*: effect of temperature.

Figure 6 shows the effect of temperature on the reaction rates in copper leaching, and for this series of experiments the variable was evaluated in the range from 289 to 323 K, keeping the pH constant at 10, $\left[S_{2}O_{3}^{-2}\right]$ = 0.5 mol/L, 750 RPM, 1 atm O_2_, and 5 g of sample. It can be seen, that as in the previous graph, the effect the values of the reaction rates were lower.

In order to determine whether our system was controlled by hydrodynamic or non-chemical variables, it was necessary to determine the activation energy, which was obtained from the treatment of *k_exp_* data according to the Arrhenius equation [52,53] where the slope obtained (m = −(E_a_/R) of the linear curve represented the activation energy (E_a_) divided by the negative value of the universal gas constant.

**Figure 7:**
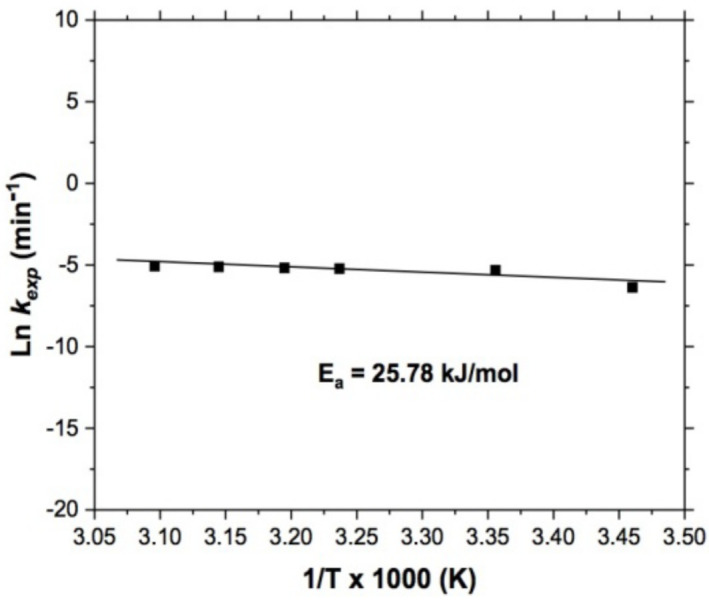
Copper leaching. Effect of temperature, E_a_ = 25.78 kJ/mol. Experimental conditions: $\left[S_{2}O_{3}^{-2}\right]$ = 0.5 mol/L, 750 RPM, pH = 10, P/P_O2_ = 1 atm and 5 g of sample.

According the above, Figure 7 shows a straight line with negative slope from which it was possible to determine the value of the activation energy. Such a slope results from plotting the Neperian logarithm of the *k_exp_* versus the inverse of the evaluated temperatures, obtaining a value of E_a_ = 25.78 kJ/mol, which indicates a mixed control (chemical and diffusive) [52]. This means that at low temperatures mass transport of oxygen at the solid−liquid interface is slow, and this controls the process, but with increasing temperature, the solid−liquid interface could become thinner, facilitating mass transport and leading to slowing of the chemical reaction.

#### 3.1.3. Effect of Stirring Rate

This effect was studied in the range of 350 to 900 RPM, keeping constant T = 318 K, pH = 10, $\left[S_{2}O_{3}^{-2}\right]$ = 0.5 mol/L, 1 atm O_2_ and 5 g of sample; the representation of the fraction of leached copper was evaluated using the kinetic model described in Equation (2) [29,30] for all experiments carried out.

**Figure 8:**
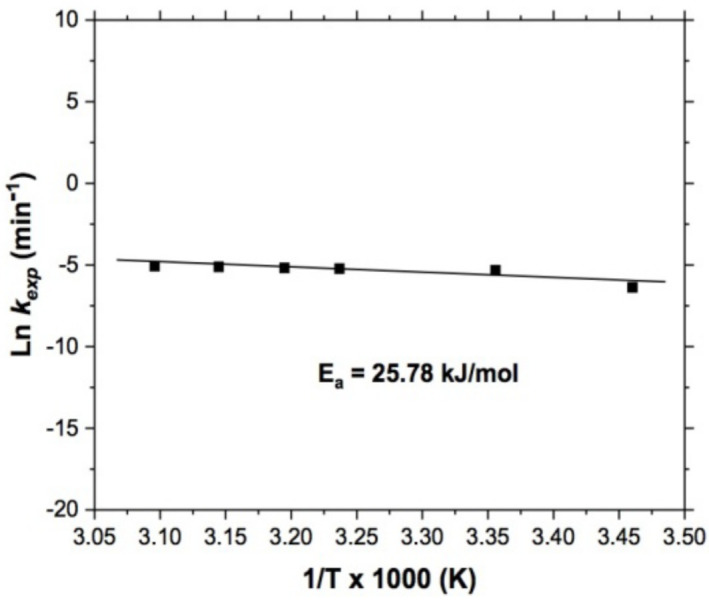
Copper leaching: effect of stirring rate.

**Figure 9:**
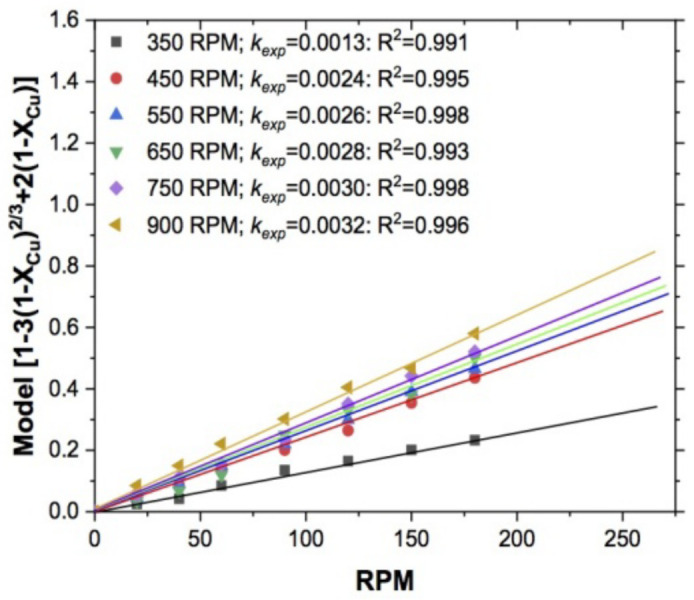
Copper leaching—effect of stirring rate. Apparent order of reaction *n* = 0.0014. Experimental conditions: 318 K, pH 10, $\left[S_{2}O_{3}^{-2}\right]$ = 0.5 mol/L, and 1 atm O_2_.

In Figure 8, the effect of stirring rate on copper leaching is shown. Note that the values obtained from the series of experiments were low, so it is important to evaluate the influence of this variable through the determination of an apparent order of reaction; for which, in Figure 9, the result of plotting the Neperian logarithm of *k_exp_* versus the RPM values is shown. The line represents the experimental rates, whose value is n = 0.0014. This value, indicates that this variable had not effect on the overall reaction rate during copper leaching, so it is necessary to evaluate other variables that complement the kinetic study and thus corroborate that the control of general reaction is of the mixed type. Also, this effect indicates that once the particles had reached a suspended condition, the chemical reaction and diffusive transport acted according to the temperature and stirring rate increases, so that the reaction went from diffusive to chemical control.

#### 3.1.4. Effect of Oxygen Partial Pressure

**Figure 10:**
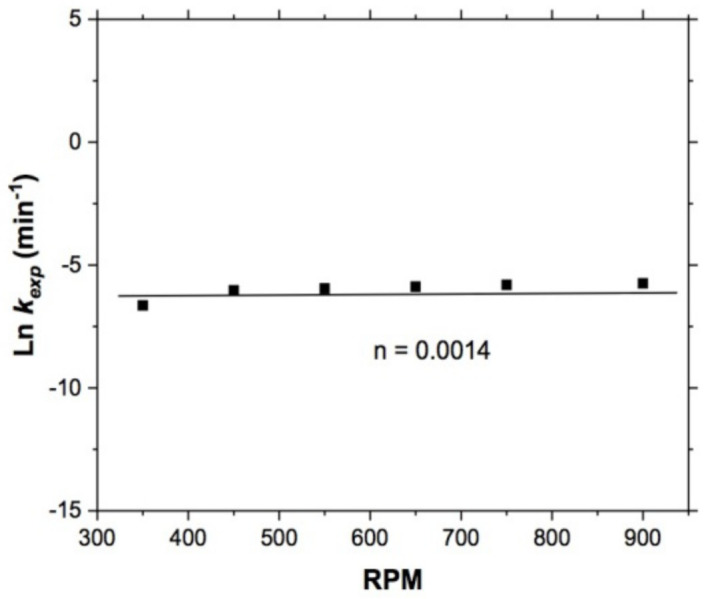
Copper leaching. T = 318 K, $\left[S_{2}O_{3}^{-2}\right]$ = 0.5 mol/L, 750 RPM, pH = 10, and 5 g of sample. Effect of oxygen partial pressure.

The effect of oxygen partial pressure was evaluated keeping the following parameters constant: T = 318 K, pH = 10, $\left[S_{2}O_{3}^{-2}\right]$ = 0.5 mol/L, 750 RPM, and 5 g of sample. The effect of this variable on the leaching of copper in the $S_{2}O_{3}^{-2}-{\;O}_{2}$ system is shown in Figure 10, where the values of the experimental constants resulting from these experiments had low values; however, it can be seen that the value of *k_exp_* at 1 atm was almost double that obtained for 0.2 atm. This result indicates that there is a strong dependence between the reaction rates and the amount of oxygen dissolved in the system.

#### 3.1.5. Effect of pH

**Figure 11:**
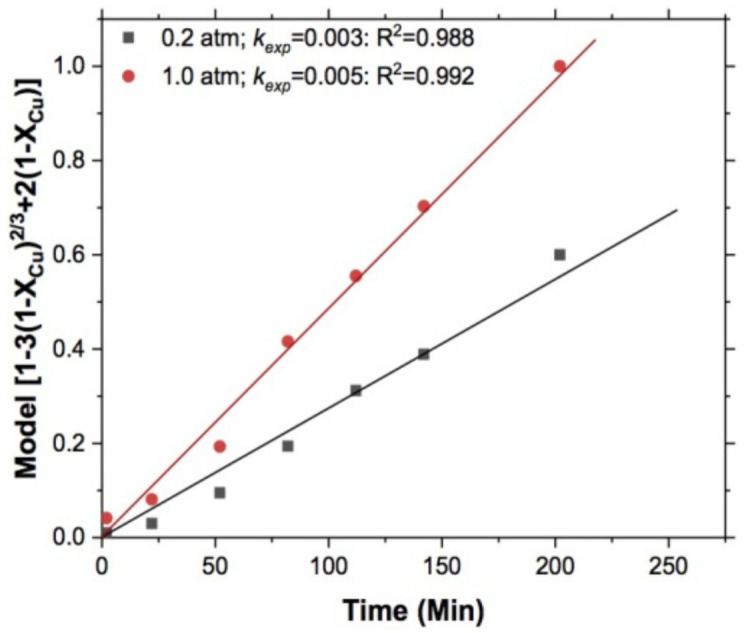
Copper leaching. Data treated with shrinking core model and diffusive control. Effect of pH.

**Figure 12:**
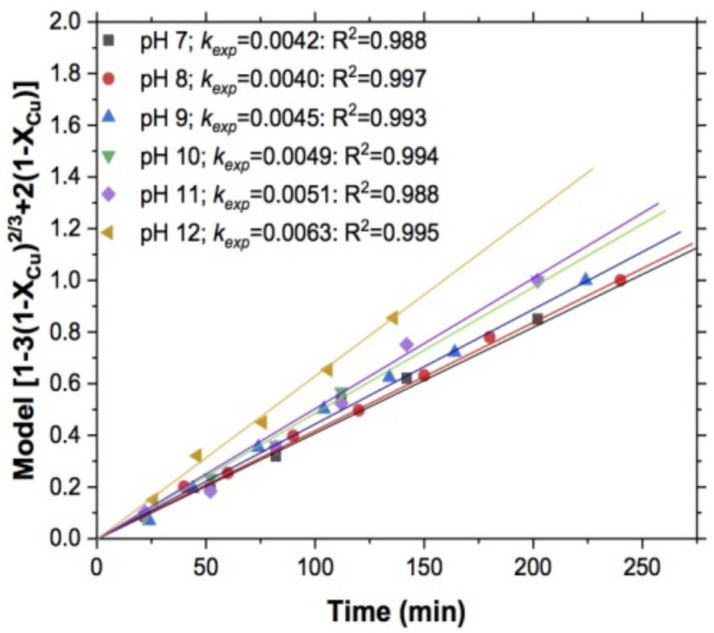
Effect of pH. Reaction order, n = 0.699. Experimental conditions: T = 318 K, $\left[S_{2}O_{3}^{-2}\right]$ = 0.5 mol/L, 1 atm O_2_, 750 RPM, and 5 g of sample.

Figure 11 shows the dependence of *k_exp_* on the pH value, and Figure 12, the effect that this variable had on copper leaching, showing a reaction order of n = 0.699. This value indicates some dependence of copper leaching on pH and that this reagent works better at high pH values, since when working at pH levels above 7 [51], the dependence of the thiosulfate on the pH is evident.

The concentration of copper in the solution increased with increasing pH, which indicates that at low values in the range studied, the oxidation of the thiosulfate was lower; likewise the oxidation of copper (I) by oxygen decreased. It was previously shown that this reaction is necessary for continued oxidation of thiosulfate; that is, with higher pH, cupric ions are reduced to cuprous, forming the cuprothiosulfate complex $(\mathrm{Cu}\left(S_{2}O_{3}\right)$ [52,54,55], showing that the pH of the system regulates the dissolution of copper, with pH between 10 and 12 being favored for our studied system. It is clear that at lower pH values and low reductive potentials, the degradation of thiosulfate will be greater, thus decreasing its concentration in solution.

## 4. Discussion

Traditionally, the thiosulfate−oxygen system in presence of Cu^2+^, has been widely used to leach precious metals such as Au and Ag. However, for the leaching of Cu, no work has been carried out using this leaching system. The most closely related works on the leaching of Cu have used ammonium and Cu^2+^, or ammonia−ammonium sulfate [37,42]. For this reason, the present work is of importance due, principally, to the use of a non-toxic reagent, which was used directly without the addition of an oxidant like HNO_3_, H_2_O_2_, O_3_, Cu^2+^, or Fe^2+^. In this process, the quick dissolution of Cu to Cu^2+^ and its complexation with thiosulfate, can avoid the thiosulfate oxidation and therefore, will promote the recovery of this value.

There has been other research related to the recovery of copper from WPCBs used acids, ionic liquids, and combinations of pyrometallurgical with hydrometallurgical and electrometallurgical processes, or even bioleaching. Acidic leaching solutions have led to the recovery of Cu, with interesting results. Some researchers also have also used nitric acid and obtained recoveries of up to 99.9% without dissolution of the gold involved, or used oxidants such as O_3_ or H_2_O_2_ [32,33,34,35]. Others have used sulfuric and hydrochloric acids to recover Cu from WPCBs and had adequate results [36,37,38].

Recently, some researchers have used a pyrolysis treatment followed by flotation to recover Cu from WPCBs, improving the recovery of Cu from 6.35% up to 96.36% [39]. In addition, bacteria have been used for bioleaching of Cu contained in these wastes [40]. Although this process is adequate environmentally, the reaction time is too long (about 12 days), but the recoveries were adequate (94% for Cu).

According the above, the leaching of copper contained in the WPCBs, has been done successfully by the thiosulfate–oxygen system, which is commonly used in the recovery of precious metals, and this system is considered as non-toxic when compared to cyaniding, and here the recovery of copper has been proven. Within this system, copper is used as an oxidizing reagent in the leaching of precious metals by adding ammonia in the leaching solution to stabilize the cupric ion and avoid the formation of the thiosulfate–copper (II) complex, which is characterized by being very stable; the formation of this complex subtracts the amount of thiosulfate present in the solution for the complexation of the precious metal of interest. Taking advantage of this feature between thiosulfate and copper (II), we decided to apply this system to WEEE, mainly in crushed PCBs; obtaining satisfactory results for the leaching of copper using the $S_{2}O_{3}^{-2}-{\;O}_{2}$ system, getting up to 99.82% of the copper. In addition to the kinetics study performed, this showed that process is governed by a mixed control (diffusive and chemical), as in Figure 5 and Figure 7. It was evident that the hydrodynamic and thermodynamic variables affected the leaching of copper, while the value of the activation energy (E_a_) was 25.78 kJ/mol, corroborating the mixed chemical–diffusive control for the leaching kinetics, which was quite similar (23.8 kJ/mol) to that obtained during the leaching of Cu from WPCBs in sulfate medium using cupric ions and oxygen [37]. This confirmed that the studied system studied is valid for leaching the Cu contained in these kind of residues, without the need to add oxidants such as HNO_3_, H_2_O_2_, Cu^2+^, or Fe^2+^.

According Rivera et al. [56], in the presence of oxygen, the reaction rate of leaching using thiosulfate, could increase. For that reason, the various reactions occurring in the copper leaching process run in parallel, and are related to the thiosulfate decomposition and could also indicate the formation of elemental sulfur (Equations (7) and (8)). This was also observed by Alymore and Muir [51]. For the case studied here, the reactions are represented by the following equations:

(6) $${\mathrm{Cu}}_{\left(s\right)}^{+2}+5S_{2}O_{3\left(\mathrm{aq}\right)}^{-2}\;\to \mathrm{Cu}{\left(S_{2}O_{3}\right)}_{3\left(\mathrm{aq}\right)}^{-5}$$

(7) $$3S_{2}O_{3}^{-2}+{\;H}_{2}O\;\to 2{\mathrm{SO}}_{3}^{-2}+4S^{0}+2{\mathrm{OH}}^{-}$$

(8) $$S_{2}O_{3}^{-2}\;\to {\mathrm{SO}}_{3}^{-2}+{\;S}^{0}$$

Then, according to the obtained results from the kinetics study (mixed control), this process could be represented by the copper leaching in the $S_{2}O_{3}^{-2}-{\;O}_{2}$ medium, as follows:

(9) r0VM[(1−3(1−XCu)23+21−XCu]dXCudt=2DePO2S2O3−2[pH]0.699× t

or

(10) r02VM [1−(1−XCu)13]dXCudt=A exp25.78PO2RT[S2O3−2]0.22[pH]0.699× t

where V_M_ = 7.09 cm^3^/mol (copper), R = 8.31 kJ/molK, T is in K, $\left[S_{2}O_{3}^{-2}\right]$ is in mol/L, r_0_ is in m, D_e_ is the diffusion coefficient through the product layer, X_Cu_ is the fraction of leached copper, t is in minutes, $P_{O_{2}}$ is the oxygen partial pressure (in atm), and 0.699 is the order of reaction for the effect of pH.

**Figure 13:**
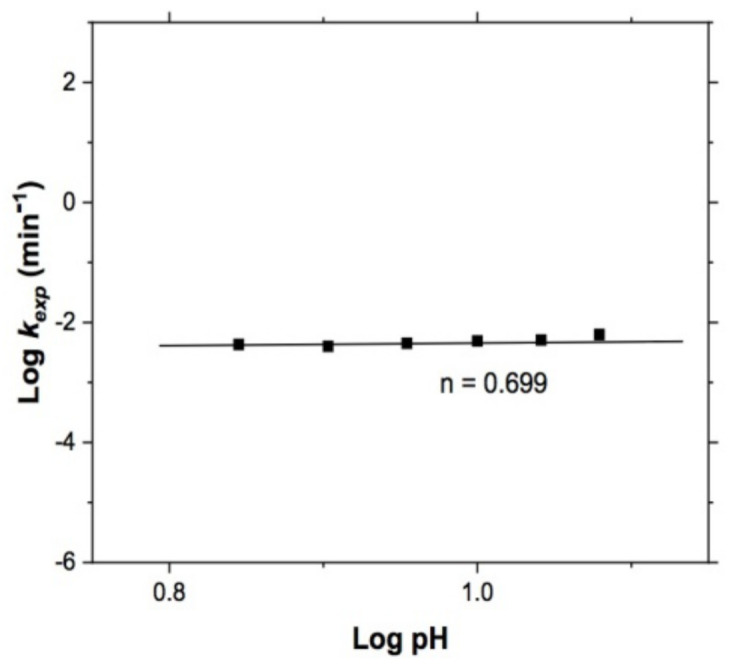
Flowsheet showing the process for the leaching of Cu contained in the WPCBs, using the thiosulfate–oxygen system.

Finally, the process of leaching of Cu contained in the WPCBs, using the thiosulfate–oxygen system is shown in Figure 13. Here, the content of copper involved in these wastes and the best conditions employed for the leaching stage can be seen. The flowsheet in Figure 13 shows that Fe and Zn were present after the leaching process, but for these metals the kinetics adjustments were not executed due these elements acting, like Cu, as oxidant reagents. However, their equilibrium constants are lower than those of Cu, so this is the reason for the low extraction values obtained for Fe (16.33%) and Zn (13.9%).

## 5. Conclusions

1. In this study, the leaching reaction according to the kinetic values found corresponded to a mixed controlled process, with, for both chemical and diffusion controls, the reaction depending of reagent concentration and temperature. The magnitude of the order of reaction found, showed a slight dependence of this variable on the reaction rate and this was also observed for the temperature, stirring rate, and pH effects.
2. Varying the temperature, it was observed that according the temperature increases, the reaction rate also increased slightly but progressively. Regularly, kinetics studies show that when there is no dependence of temperature during leaching, it is because the rate is controlled by diffusion and in the contrary case the rate is controlled by chemical reaction. In this case, due to the poor effect of temperature, both cases could be acting, giving a mixed control of reaction, which was confirmed by the activation energy found, E_a_ = 25.78 kJ/mol.
3. In the case of stirring rate, the results indicated that once particles had reached a suspended condition, both chemical reaction and diffusion could begin smoothly, and here the speed of agitation appeared to have a very poor effect on copper leaching.
4. The effect of oxygen partial pressure indicated the marked influence of this variable on the copper leaching rate. The effect of O_2_ can also help to determine if the process improves with a more oxidizing or more reducing environment, and depending of the amount of dissolved oxygen into the solution, rapid oxidation of Cu (I) to Cu (II) occurs with some oxidation of the thiosulfate to produce sulfate and trithionate. An excess of oxygen is detrimental for the extraction of gold, but beneficial for the oxidation of thiosulfate and the its complexation with Cu; the main role of excess of oxygen is to degrade thiosulfate, possibly via di-sulfite $\left(S_{2}O_{5}^{-2}\right)$. The rapid rate reduction of copper (II) by thiosulfate compared to the slow oxidation of Cu (I) by oxygen allows to the determination of the rate of oxidation of thiosulfate using steady state kinetics.
5. The effect of pH showed that the thiosulfate was stable within the range of pH values evaluated and also, that this reagent works better at alkali pH values. In addition, it was expected that this effect would not influence the copper-leaching rate, since as was observed in the Eh - pH diagram [56], thiosulfate is stable in the evaluated range. In the case of our study, according to the pH range studied, the pH of solution working under oxidizing conditions, and the formation of water-soluble metal sulfates (in this case, Cu) was facilitated by the presence of metal cations. At low pH, the amount of H^+^ ions is greater, so that many are lodged in the active sites of the leaching reagent, limiting the absorption of metal ions as the pH increases, and the disposition of these sites increases. This is why the leaching rate of Cu was not strongly dependent on pH values, and was found to be related to the mixed control.

## Figures and Tables

**Table 1:** Concentration of metals present in the WPCBs.

Element	mg/g
Copper (Cu)	744.2
Iron (Fe)	24.75
Zinc (Zn)	22.21

**Table 2:** Experimental conditions used for the study of copper leaching.

Parameters	Experimental Conditions
$\left[S_{2}O_{3}^{-2}\right]$ (mol/L)	0.075, 0.1, 0.2, 0.4, and 0.5
pH	7, 8, 9, 10, 11, and 12
Sample weight (g)	5.0
Temperature (K)	289, 298, 308, 313, 318, and 323
Oxygen partial pressure (pure O_2_) (atm)	0.2 and 1.0
Stirring rate (RPM)	350, 450, 550, 650, 750, and 900

## Data Availability

Not applicable.
